# Impact of Social Deprivation on Cubital Tunnel Syndrome Treatment Timeline

**DOI:** 10.1016/j.jhsg.2024.08.019

**Published:** 2024-09-26

**Authors:** Akhil Dondapati, Janet Ngoc Tran, Callista Zaronias, Cody C. Fowler, Thomas J. Carroll, Bilal Mahmood

**Affiliations:** ∗ Department of Orthopaedic Surgery, University of Rochester Medical Center, Rochester, NY; † University of Rochester Medical School. Rochester, NY

**Keywords:** Area deprivation index, Cubital tunnel release, Cubital tunnel syndrome, Risk factors, Socioeconomic status

## Abstract

**Purpose:**

The purpose of this study was to establish the impact of area deprivation index (ADI) on treatment timelines of patients with cubital tunnel syndrome (CuTS). We hypothesize that increased social deprivation will correlate with increased time between care milestones from presentation to surgery.

**Methods:**

This is a retrospective study of patients diagnosed with CuTS who underwent surgical intervention at a single academic institution. Variables including age, sex, body mass index, ADI, electrodiagnostic (EDX) severity classification, and time elapsed between treatment milestones were obtained. Treatment milestones included time elapsed between initial presentation to hand surgery and EDX studies, and surgery. Analysis included univariate χ^2^ tests and analysis of variance, as well as multivariate linear and logistic regressions.

**Results:**

Six hundred and fifty-three patients were divided by ADI national percentiles from the least to most deprived tertiles. Univariate analysis found no differences in care timelines across ADI tertiles. Multivariate analysis revealed a nonsignificant trend toward higher ADI predicting longer time from presentation to surgery. Moderate EDX rating correlated with increased time from presentation to surgery. Mild EDX ratings correlate with increased time from EDX studies to surgery. Age was a significant predictor of decreased time between initial presentation and surgery and between EDX and surgery. Completion of EDX studies prior to presentation significantly decreased time to surgery.

**Conclusions:**

Social deprivation does not significantly correlate with delays in the treatment timeline for CuTS. Increased age was significantly correlated with shorter treatment timelines, which may reflect differences in physicians’ approaches to patients of different ages. Electrodiagnostic testing obtained prior to initial presentation expedited care following presentation to hand clinic. However, this could reflect either an overall delay in care (if EDX were obtained because of a delay from referral to presentation) or truly expedited care.

**Type of study/level of evidence:**

Prognostic II.

Cubital tunnel syndrome (CuTS), the second most common upper-extremity compressive neuropathy, has a prevalence of approximately 6% and an incidence of 30 per 100,000 person-years.^1^, ^2^, ^3^ Symptoms at initial presentation can vary but will often begin with intermittent pain, paresthesias, and dysesthesias in an ulnar nerve distribution and can progress to intrinsic muscle atrophy and weakness.^4^ Diagnosis can be clinical but is often supplemented with studies including ultrasound and electrodiagnostic (EDX) studies.^5^, ^6^, ^7^ Treatment can start with activity modification and bracing but will often progress to requiring cubital tunnel release (CuTR).^8^ Cubital tunnel release incidence has been increasing, with the number of cases increasing by 47% from 1996 to 2006.^9^ Given this increase, it is important to identify factors that may be influencing a patient’s ability to access this treatment modality.

Many factors can affect both pre- and postoperative outcomes of patients with CuTS, including body mass index (BMI), presence of comorbidities, age, and severity on EDX.^10^, ^11^, ^12^ Socioeconomic status (SES) is one such factor, incorporating income, patient education level, occupation, and more. This has been known to affect health care access, patient perceptions of their care, and patient-reported outcomes.^13^, ^14^, ^15^ Within orthopedic literature, SES effects on the total joints and trauma populations have been the most well studied.^16^ In hand surgery patients, social deprivation has been shown to affect carpal tunnel syndrome prevalence. Carpal tunnel syndrome is more prevalent in populations with a lower SES.^17^ These patients also have higher rates of psychological impact, bilateral disease, and other comorbidities; however, data regarding patient-reported outcomes have been mixed.^17^^,^^18^ Fewer studies about the impact of SES on CuTS exist, but they established that those with greater social deprivation have higher rates of surgery.^19^^,^^20^ Having a lower education level has also been shown to be a risk factor for ulnar nerve entrapment.^21^

Social deprivation refers to a lack of access to opportunities and resources necessary for social wellbeing. A validated measurement of social deprivation, the area deprivation index (ADI), can capture disparities within the orthopedic population.^22^ The ADI factors in socioeconomic disadvantage within specific neighborhoods. Because of the previously established importance of SES on patient health, the literature on ADI is expanding. With CuTS specifically, however, ADI has been minimally investigated. Rogers et al^23^ demonstrated no significant difference in patient expectations of improvement after CuTR based on their ADI but did not investigate any patient-reported outcomes or delays in health care access. Two studies have looked at the distressed communities index (DCI), with one showing no association between DCI and symptom duration, time from presentation to CuTR, and presenting CuTS severity, whereas the other demonstrated a greater risk of disease severity with a higher DCI.^24^^,^^25^ The DCI is similar to the ADI; however, ADI has a greater capacity to be more granular than DCI.^26^

Although ADI and social deprivation are established influences on health care outcomes and access, there remains little and mixed literature regarding their effects on CuTS. The purpose of this study was to establish the impact of ADI on treatment timelines of patients undergoing CuTR. We hypothesize that increased social deprivation will correlate with increased time between care milestones from presentation to surgery.

## Methods

A retrospective review of adult (≥18 years of age) patients who underwent cubital tunnel surgery at a single institution between 2016 and 2023 was conducted. This study was approved by our institutional review board. Consent was waived by the institutional review board because of the retrospective nature of the study. We identified 1,893 patients using the current procedural terminology code 64718. The study cohort of 653 patients was chosen by randomized selection. Exclusion criteria applied to any patient with treatment related to trauma, a contralateral diagnosis, or a revision surgery. Patient demographics, baseline characteristics, EDX study results, timing of CuTS diagnosis, presentation to hand surgery to EDX or CuTR, and timing of EDX to surgery were reviewed from the electronic medical record and compared. Electrodiagnostic severity was classified as mild, moderate, or severe and was determined by the electrophysiologist who performed the examination. We used the national ADI to assess the social deprivation of study subjects. Area deprivation index is a measure of socioeconomic disadvantage developed by Kind et al,^27^ which is validated to the Census block group level and represents the degree of social deprivation as a percentile, with higher ADI indicating greater deprivation. Patient zip codes were obtained via chart review in order to identify their respective national ADI percentile.

### Statistical analysis

The overall cohort was stratified into three tertiles by ADI percentile. We used descriptive statistics including proportions, medians, and interquartile ranges to summarize data for the overall population and each subgroup. Univariate analysis of categorical and continuous variables was performed with χ^2^ tests and analysis of variance, respectively. Post hoc pairwise comparisons of time between care milestones were performed with Tukey’s tests. Multivariate linear regression analyzed factors impacting the time between care milestones. We treated ADI as a continuous variable for multivariate analysis. Statistical significance was defined as *P* ≤ .05. All analyses were performed using Stata 18 (StataCorp).

## Results

A total of 653 patients were included in the study. Three ADI groups were established based on tertiles. There were 344 patients in the highest tertile, 265 in the middle, and 44 in the lowest. The average age of the patients was 56 years old. The groups had 62.2% men with an average BMI of 31.7. No differences were found in the baseline demographics between the groups with regard to age, sex, BMI, thyroid disease, or diabetes. Additionally, there were no significant differences in the number of patients with EDX prior to initial presentation, EDX severity, or likelihood of receiving a steroid injection between the cohorts (*P* > .05). Patients in the highest ADI tertile, however, had more patients that were Black (*P* < .001), Hispanic (*P* = .029), and smokers (*P* = .03; Table 1).

**Table 1:** Patient Characteristic Comparisons Based on National ADI Tertiles∗

Characteristic	Overall	Low Tertile ADI (1–33)	Middle Tertile ADI (34–66)	High Tertile ADI (67–100)	*P* Value
n	653	44	265	344	
Age (y)	56.0 ± 14.1	58.3 ± 14.9	56.9 ± 14.4	55.0 ± 13.7	.14
M (%)	406 (62.2)	28 (63.6)	173 (65.3)	205 (59.6)	.349
BMI (kg/m^2^)	31.7 ± 6.8	32.0 ± 6.4	31.6 ± 7.0	31.7 ± 6.7	.924
Race (%)					< .001†
White	544 (83.3)	37 (84.1)	247 (93.2)	260 (75.6)	
Black	84 (12.9)	3 (6.8)	11 (4.2)	70 (20.4)	
Other	25 (3.8)	4 (9.1)	7 (2.6)	14 (4.1)	
Hispanic ethnicity (%)	21 (3.2)	1 (2.3)	3 (1.1)	17 (4.9)	.029†
Thyroid disease (%)	75 (11.5)	8 (10.2)	29 (10.9)	38 (11.1)	.353
Smoking (%)					.03†
Active	116 (17.8)	3 (6.8)	38 (14.3)	75 (21.8)	
Former	234 (35.8)	15 (34.1)	102 (38.5)	117 (34.0)	
Never	303 (46.4)	26 (59.1)	125 (47.2)	152 (44.2)	
Diabetes (%)	167 (25.6)	11 (25.0)	62 (23.4)	94 (27.3)	.543
EDX severity (%)					.699
Mild	137 (21.4)	11 (25.6)	56 (21.5)	70 (20.7)	
Moderate	104 (16.2)	7 (16.3)	44 (16.9)	53 (15.7)	
Severe	142 (22.2)	8 (18.6)	57 (21.9)	77 (22.8)	
Steroid injections (%)	30 (4.6)	2 (4.6)	10 (3.8)	18 (5.2)	.695
Time from hand presentation to EDX (days)	34.5 (20, 54)	28.5 (19, 48)	30 (20, 49)	37 (21, 56.5)	.452
Time from EDX to surgery (d)	91.5 (46, 219.5)	88 (45, 166)	90 (46, 214)	96 (47, 229)	.191
Time from hand presentation to surgery (days)	95 (49, 176)	78.5 (42.5, 138)	87 (48, 162)	99.5 (50, 198)	.11
EDX prior to presentation (%)	240 (36.8)	13 (29.6)	102 (38.5)	125 (36.3)	.508

EDX, electrodiagnostic studies.

∗ Data are presented as number (%), median (interquartile range), and mean ± standard deviation as appropriate.

† *P* < .05.

On univariate analysis, there were no differences noted in the number of days from initial presentation to EDX (34.5 days vs 28.5 days vs 30 days, *P* > .05), time from EDX to surgery (91.5 days vs 88 days vs 90 days, *P* > .05), or days from presentation to surgery (95 days vs 78.5 days vs 87 days, *P* > .05) between the lowest, middle, and highest ADI tertile (Table 1).

Multivariate analysis demonstrated age correlating with a decreased time between both initial presentation to surgery (−3.0 ± 1.8 days, *P* = .001) and EDX to surgery (−2.5 ± 1.8 days, *P* = .008). Completion of EDX prior to presentation also significantly decreased the time from presentation to surgery (−94.4 ± 47.9 days, *P* < .001). With regard to EDX severity, patients with mild EDX had increased time from EDX to surgery (152.4 ± 69.3 days, *P* < .01), and those with moderate EDX had increased time from presentation to surgery (111.9 ± 69.0 days, *P* = .02). There was a nonsignificant trend toward higher ADI predicting longer time from initial presentation to surgery (*P* = .091). Full multivariate analyses can be seen in Table 2.

**Table 2:** Time Between Initial Evaluation and Electrodiagnostic Studies to Surgery: Multivariate Analysis

	Time From Hand Presentation to Surgery			Time From EDX to Surgery		
	Coefficient	95% CI	*P* Value	Coefficient	95% CI	*P* Value
ADI national percentile	1.077	(−.174, 2.329)	.091	0.946	(−.351, 2.243)	.152
Age	−3.033	(−4.834, −1.232)	.001∗	−2.500	(−4.338, −.661)	.008∗
M	−0.800	(−50.667, 49.067)	.975	1.345	(−49.528, 52.217)	.959
BMI (kg/m^2^)	−1.213	(−4.735, 2.309)	.499	−2.598	(−6.210, 1.015)	.158
White	37.973	(−91.724, 167.670)	.566	27.898	(−104.190, 159.986)	.678
Black	36.389	(−107.540, 180.318)	.62	−12.408	(−159.125, 134.309)	.868
Hispanic	4.607	(−134.934, 144.147)	.948	34.349	(−107.682, 176.381)	.635
Thyroid disease	48.253	(−24.624, 121.129)	.194	26.286	(−48.311, 100.882)	.489
Diabetes	−16.314	(−71.456, 38.827)	.561	−24.487	(−81.041, 32.067)	.395
EDX prior to presentation	−94.402	(−142.327, −46.477)	<.001∗	43.490	(−18.720, 105.700)	.17
Mild EDX	51.203	(−9.743, 112.149)	.099	152.345	(83.016, 221.674)	<.001∗
Moderate EDX	111.934	(42.865, 181.002)	.002∗	40.581	(−23.731, 104.894)	.216
Severe EDX	20.020	(−43.031, 83.070)	.533	−37.904	(−146.968, 71.160)	.495

CI, confidence interval; EDX, electrodiagnostic studies.

∗ *P* < .05.

## Discussion

Despite the increased awareness of the effect of social deprivation and ADI in the hand surgery population, its influence on patients with CuTS has yet to be elucidated. Our study demonstrated no significant differences in the baseline demographics between ADI tertiles except for race and smoking status (*P* < .05). Although there was a trend to a relationship between time to surgery and ADI, it was ultimately nonsignificant, and thus, ADI itself did not influence delays in care. Age, as well as the presence of EDX studies prior to presentation, were correlated with shorter treatment timeline to surgery. Additionally, those with mild or moderate EDX severity demonstrated increased time to surgery.

Previous studies investigating social factors and SES demonstrated that CuTS patients who were publicly insured had delays in orthopedic evaluation and more severe initial clinical and EDX findings compared to those with private insurance.^28^ Additionally, Zimmerman showed higher rates of CuTR in patients with lower SES, although outcomes largely did not seem to differ.^20^ Lower educational level also appears to have a higher risk of ulnar nerve compression (odds ratio: 1.45), in addition to smoking status (odds ratio: 2.94).^21^ In our study, those in the highest ADI tertile were more likely to be smokers. Although our study did not determine prevalence levels, we did not find any differences in EDX severity between the groups. If there was truly an increased prevalence of CuTS in smokers in our cohort, this relationship does not appear to influence EDX studies and, perhaps, may more likely be because of the presence of solely clinical symptoms.

Using the index of multiple deprivation, patients with greater social deprivation had higher rates of CuTR at a younger age in the United Kingdom.^19^ Most other studies investigate the DCI as a measure of social deprivation. Zhang looked at CuTR patients and found worse EDX severity in patients with higher DCIs.^25^ Grisdela et al,^24^ on the other hand, demonstrated no significant association between DCI and patient-reported symptom duration, time from presentation to surgery, or severity of CuTS (*P* > .05). Area deprivation index and DCI differ with regard to how they are collected. Distressed communities index is determined by zip codes, whereas ADI is measured at the block level, allowing it to potentially be more granular.^26^^,^^29^ Our results were similar to Grisdela, as we did not find delays in care of CuTS based on ADI. Although the correlation between social deprivation and CuTR outcomes has not been investigated, there are a number of studies establishing this relationship in various hand surgery conditions.^17^^,^^30^^,^^31^ Our study indicates that if this relationship extends to CuTR, these potentially worse outcomes are not related to delays in care.

When looking into social factors, we found that older age was correlated with expedited time to surgery, both from initial presentation and from EDX completion. Our results align with Day et al,^32^ who studied predictors of CuTR patients and found that age was the most important predictor of ulnar nerve surgical release. Patients were grouped into three age categories (18–44 years old, 45–64 years old, and ≥65 years old) and had respective surgical rates of 28%, 57%, and 66%.^32^ This potentially reflects a surgeon attempting to trial nonsurgical management for longer in younger patients prior to considering surgical intervention. Despite Johnson showing higher rates of CuTR in younger patients, these patients were in more deprived quintiles, which may have been a confounding factor.^19^ Delays in surgery have not been shown to correlate with sex and race, although it may be associated with publicly insured patients.^24^^,^^28^ Similarly, our results also demonstrated no significant correlation between sex, race, or ethnicity with delays in care (*P* > .05).

With regard to EDX, we observed that patients with mild or moderate EDX studies had significantly longer times to surgery than those with severe EDX (*P* < .05). Many surgeons will use EDX to supplement decisions about pursuing surgical intervention in CuTS patients.^33^ This likely reflects the decision making observed in our study, as our surgeons may want to expedite CuTR in those with severe EDX. Additionally, we found that obtaining EDX prior to presentation led to fewer days to surgery from the initial presentation at hand surgery clinic. This can be interpreted in one of two ways. First, it may reflect an overall delay in care if EDX were obtained by the primary care physician (PCP) because of a significant delay from the time of referral placement to the initial hand surgery presentation. Second, it may truly show expedited care if a patient has more resources to obtain EDX quickly or has short times from PCP referral placement to EDX testing. Despite both these conclusions being in direct contrast to one another, it is difficult to know which is more accurate. Further studies may seek to establish time from PCP referral to EDX and, subsequently, hand surgery treatment to clarify this relationship.

Our study has several notable limitations. First, it was a retrospective review and is prone to selection bias. The study only included patients who underwent surgical intervention for CuTS. There may have been some patients who were indicated for surgery but were not able to receive it because of resource availability or cost. Since the surgery is often an elective procedure, there may have been delays in the time to surgery because of other confounding factors such as a patient’s desire to have surgery during a specific timeframe. This study’s least deprived cohort has fewer patients than the other cohort, which may yield some bias. All subjects in our study are from a single academic institution in New York State; hence, our results may not be generalizable to other populations. Additionally, we were not able to account for an inaccurate ADI if a patient’s address was incorrect. Additionally, although ADI is validated, it may not completely reflect a patient’s SES as it is an estimate.

In summary, although previous studies have correlated social deprivation with poorer outcomes in hand surgery patients, we found that social deprivation does not significantly correlate with delays in the treatment timeline for CuTS. Future multicenter studies are required to better define the relationship between social deprivation and CuTS treatment.

## Conflicts of Interest

No benefits in any form have been received or will be received related directly to this article.
